# Lessons Learned During the Pandemic: Correlation of QT Intervals Between Telemetry and 12-Lead Electrocardiogram

**DOI:** 10.7759/cureus.16877

**Published:** 2021-08-04

**Authors:** Mohamed Farhan Nasser, Ahmad Jabri, Ashish Kumar, Saima Karim, Elizabeth S Kaufman

**Affiliations:** 1 Heart and Vascular Center, MetroHealth Medical Center/Case Western Reserve University, Cleveland, USA; 2 Department of Critical Care Medicine, St. John's Medical College, Bangalore, IND

**Keywords:** 12-lead ecg, telemetry, abnormal repolarization, prolonged qt, qt interval

## Abstract

Background and objective

QT prolongation is associated with an increased risk of ventricular arrhythmias. Since some patients on contact or droplet precautions require QT-prolonging medications, monitoring the QT interval may become imperative to prevent fatal arrhythmias. To limit the exposure of staff to patients during and even after the coronavirus disease 2019 (COVID-19) pandemic and judiciously use personal protective equipment (PPE), it is important to find alternatives to frequent 12-lead electrocardiograms (ECG). The objective of this study was to compare QT intervals measured on telemetry to those measured on 12-lead ECG to determine whether telemetry QT interval measurements could be used in place of 12-lead measurements.

Methods

Simultaneous telemetry recordings via a Philips telemetry monitoring system (Philips Healthcare, Eindhoven, Netherlands) and 12-lead ECGs were obtained from 50 patients. Patients were from cardiac telemetry and cardiac intensive care units. QT interval from the telemetry system was compared to the QT interval on the 12-lead ECG. QT intervals on two telemetry strips were uninterpretable as the termination of the T-wave could not be defined appropriately; therefore, these patients were excluded.

Results

In 33 of 48 patients (69%), QT intervals from the telemetry studies matched the QT intervals measured by 12-lead ECG. The intraclass correlation coefficient (ICC) between telemetry QT and 12-lead ECG QT was 0.887 (95% CI: 0.809-0.934; p<0.001). In 15 of 48 patients (31%), the QT intervals measured from telemetry were different from those measured by 12-lead ECG. These patients either had an abnormal rhythm, conduction abnormalities, or repolarization abnormalities at baseline.

Conclusion

Telemetry is a suitable alternative for measuring QT intervals in the majority of patients. However, those with baseline ECG abnormalities should have serial 12-lead ECGs. This can reduce the risk of staff exposure to pathogens and prevent overuse of PPE during the COVID-19 pandemic and for other patients in isolation.

## Introduction

Prolonged QT interval is associated with an increased risk of fatal ventricular arrhythmias and sudden cardiac death. QT interval is ideally measured from a 12-lead electrocardiogram (ECG); however, telemetry measurement may suffice in specific situations. Occasionally, there are situations when isolation precautions need to be followed, and reliance on telemetry to measure QT intervals may be needed along with arrhythmia interpretation.

A prolonged QT interval is defined as 460 milliseconds (ms) or longer in women and 450 ms or longer in men [1]. Previous guidelines have recommended that one of the ECG limb leads should be used to measure QT, but in patients with drug-induced long QT syndrome, the most accurate measurement is obtained by using both limb and precordial leads [2].

Before and during the coronavirus disease 2019 (COVID-19) pandemic, many patients on contact or droplet precautions have been started on QT-prolonging medications or anti-arrhythmic medications and require frequent monitoring of their QT intervals [3]. While measuring and monitoring the QT interval is crucial in preventing ventricular arrhythmias, it is also important to limit the exposure of staff members to patients and conserve personal protective equipment (PPE) [4]. To protect healthcare personnel and conserve supplies while providing appropriate care, we tested the hypothesis that a QT interval measured from telemetry recordings would be an adequate alternative to that measured from a 12-lead ECG.

This article was previously presented as a meeting abstract at the 2020 annual American Heart Association meeting in November 2020.

## Materials and methods

We retrospectively obtained and printed simultaneous resting 12-lead ECG and telemetry recordings from 50 adult patients admitted to the cardiac intensive care unit or cardiac telemetry unit at MetroHealth Medical Center in Cleveland, Ohio. All patients used a Philips Healthcare telemetry monitoring system (Philips Healthcare, Eindhoven, Netherlands).

The QT intervals were measured manually using calipers via the tangent method: a tangent was drawn from the peak of the T-wave through the downslope, and the intersection between the tangent and the isoelectric line was marked as the end of the T-wave [5,6]. The 12-lead ECGs and telemetry strips were printed out and manually measured. Two clinicians made all measurements independently, and discrepancies were resolved through consensus. Clinicians who made the measurements were an electrophysiologist and a general cardiology fellow in training. The readers were blinded to the patients' names and their respective telemetry and 12-lead ECGs to reduce bias. As a result, ECGs and telemetry were evaluated in a scrambled order, minimizing the chance that the same patient’s recordings would be read sequentially.

Telemetry recordings included two to three simultaneous leads from lead I, lead II, and a precordial lead. QT interval was measured using both limb and precordial leads. For every patient, the most prolonged QT from telemetry was compared to the most prolonged QT measured on 12-lead ECG. In cases of atrial fibrillation (AF), we measured multiple QT intervals and calculated the means across all the complexes [7]. The heart rates on both the 12-lead intervals and telemetry strips were identical because they were taken simultaneously. Therefore, QT did not require correction for heart rate.

After assessing the distribution using a quantile-quantile plot, numerical variables were presented as the median and interquartile range (IQR), while categorical variables were presented as numbers. Numerical variables were compared using the Mann-Whitney U test while categorical variables were compared using the chi-square test. Interrater reliability (IRR) of telemetry and 12-lead ECG measurements of QT intervals were assessed using intraclass correlation coefficient (ICC) and corresponding 95% CI. Further, to report the correlation between telemetry and 12-lead ECG-measured QT intervals, we used ICC and corresponding 95% CI. We used a “one-way model agreement” with a single unit of analysis to compute the ICC. The p-value for ICC that was significantly different from 0 was computed. We used IRR package Version 0.84.1 in R Version 3.6.1 (R Development Core Team) to analyze the results.

## Results

Patient characteristics

Of the 50 patients, 17 were women (34%), and 33 were men (66%). The median age was 65 years (IQR: 57.2-71). Twenty-eight patients (56%) had conduction abnormalities and/or arrhythmias: five (10%) were in AF, five (10%) were in atrial flutter (AFL), two (4%) were in junctional rhythm, two (4%) were in complete heart block (CHB), one (2%) was in multifocal atrial tachycardia (MAT), one (2%) had ectopic atrial rhythm, and three (6%) were ventricularly paced (VP). Right bundle branch block (RBBB) was present in nine patients (18%), left bundle branch block (LBBB) in three patients (6%), and nonspecific intraventricular conduction delay (IVCD) was present in one patient (2%). Most of the patients (41 of 50) were tested for COVID-19 and returned a negative result; the remaining patients were not tested for COVID-19.

QT interval measurements

**Figure 1:**
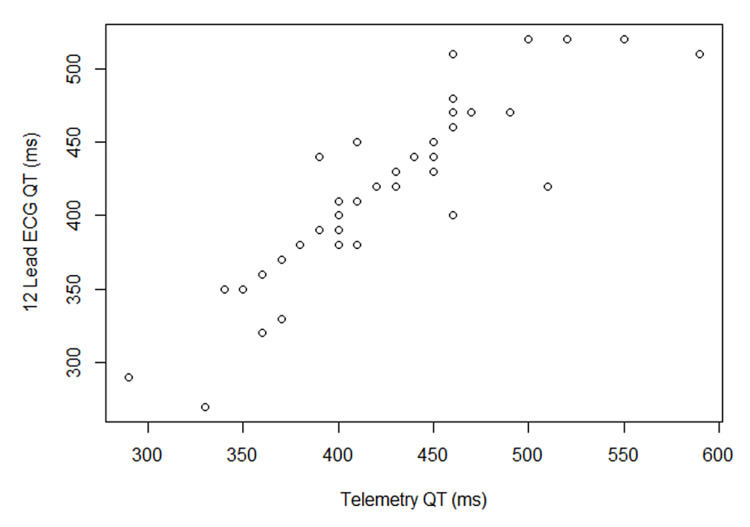
Graphical representation of the telemetry and 12-lead ECG QT intervals Multiple patients with a similar combination of 12-lead ECG and telemetry measured QT intervals were only represented once on the graph ECG: electrocardiogram; ms: milliseconds

**Figure 2:**
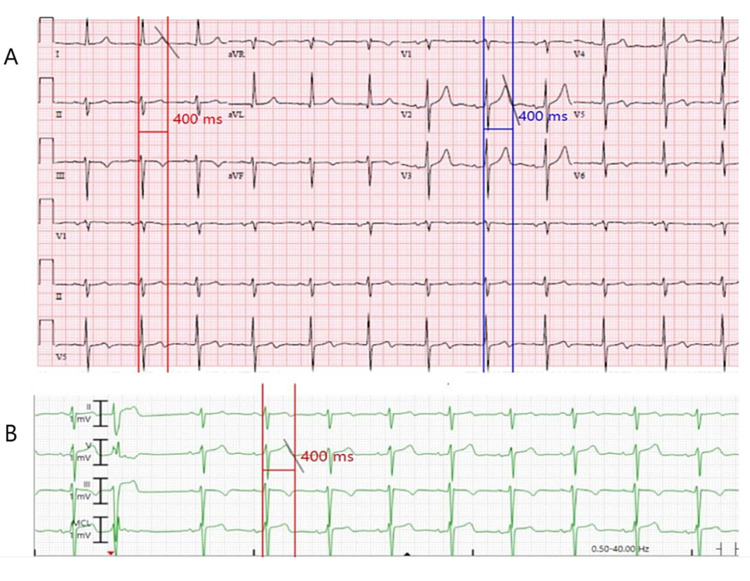
12-lead ECG (A) and telemetry (B) for a patient showing a QT-interval of 400 ms These measurements were initially made manually by both readers after the 12-lead ECG and telemetry strips were printed. Similar measurements are shown with digital calipers ECG: electrocardiogram; ms: milliseconds

QT intervals on two telemetry strips were uninterpretable as the termination of the T-wave could not be defined appropriately; therefore, these patients were excluded. One of these patients had AFL, and both had RBBB. Overall, measurements between the two readers matched within 10 ms in all patients. The QT intervals measured from telemetry matched those measured by 12-lead ECG within 10 ms in 33 patients (69%). The ICC for interrater telemetry QT measurement was 0.973 (95% CI: 0.953-0.985; p<0.001). The ICC for interrater 12-lead ECG QT measurement was 0.943 (95% CI: 0.902-0.967; p<0.001). The ICC between telemetry-measured QT and 12-lead ECG-measured QT was 0.887 (95% CI: 0.809-0.934; p<0.001), signifying a good correlation. Figure 1 is a graphical representation of the measured QT intervals showing a good correlation between telemetry and 12-lead ECG when the QT interval was in the 350-480 ms range. Figure 2 is a pictorial representation of a patient with concordant QT intervals between 12-lead ECG and telemetry.

In 15 patients (31%), the QT measurements between telemetry and 12-lead ECG were discordant by 20 ms or more. The median difference was 40 ms (IQR: 20-50). Of these 15 patients, rhythm or conduction abnormalities were present in 13 (87%) and abnormal repolarization in two (13%).

Of all the patients with a normal 12-lead ECG, 19 of 21 (90%) had matching QT intervals, whereas in patients with abnormalities, 14 of 27 (52%) had matching QT intervals: three of seven patients (43%) with RBBB, one of one (100%) with IVCD, one of five (20%) with AF, two of two (100%) with junctional rhythm, two of two (100%) with CHB, three of three (100%) with VP, one of one (100%) was in ectopic atrial rhythm, and two of two (100%) with abnormal repolarization had discordant QT intervals. All patients with LBBB, MAT, and AFL had matching QT intervals. 

## Discussion

Very few studies have compared QT measurements from telemetry with those from 12-lead ECGs. One study has revealed that QT intervals were longer when measured on telemetry than a 12-lead ECG [8]. Rimmer et al. showed that lead I or lead II telemetry measurements correlated best with 12-lead ECG readings, but they recommended obtaining 12-lead ECGs in patients with prolonged QT intervals. Another recent study by Braunstein et al. used a mobile patch telemetry system to successfully detect arrhythmias and monitor the QT interval [9].

We found that telemetry served as an adequate surrogate for the 12-lead ECG to assess QT interval in most patients. Specifically, telemetry provided adequate QT interval measurements in patients with normal baseline 12-lead ECG. In contrast, in approximately half of the patients with an abnormal ECG, QT measurements between telemetry and 12-lead ECGs were discrepant, and in these patients, telemetry did not serve as an adequate alternative to assess the QT interval. The results of our study also show that there was not a good correlation in patients with prolonged QT >500 ms, thereby highlighting the importance of repeating a 12-lead ECG to follow QT interval in these patients.

In patients with baseline ECG abnormalities such as abnormal depolarization or repolarization, there tends to be a greater dispersion in the measured QT intervals between the leads on the 12-lead ECG [1]. Since telemetry only records a limited number of these leads, QT interval may be discrepant from the ECG because the lead with the most prolonged QT interval may not be represented on telemetry. Additionally, in patients with other baseline abnormalities such as AFL or CHB, it can be challenging to determine which signals are part of the T-wave, and consequently, the QT interval because of the limited number of leads available for review. Relying solely on telemetry in these situations can lead to inaccurate readings.

Based on our findings, we suggest checking a baseline 12-lead ECG on every patient to evaluate for abnormal rhythms, abnormal conduction, or abnormal repolarization. If there is a significant abnormality on the baseline ECG, the QT interval should be followed with serial 12-lead ECGs. In the absence of these abnormalities, telemetry can serve as an adequate surrogate to monitor for prolongation of the QT interval.

Limitations

Our study was limited by its small sample size. Our results need to be studied and validated in a larger and more diverse population. Secondly, it may also be reasonable to perform this study using other telemetry systems as well. Finally, we did not account for telemetry technician experience, which could have played a major role in achieving a high correlation between telemetry and 12-lead ECG-measured QT.

## Conclusions

Telemetry recordings can serve as a useful alternative to follow the QT interval in patients without abnormalities at baseline. Of patients with a normal baseline ECG, the vast majority had an accurate correlation of QT interval, while approximately half of the patient population with an abnormal baseline ECG had QT intervals correlate between the ECG and telemetry.

For patients on contact, droplet, or airborne precautions, minimizing serial 12-lead ECGs can reduce the risk of exposure of healthcare workers and preserve PPE. While our findings have an immediate utility during the current state of the COVID-19 pandemic, they will continue to be useful if patients are on contact precautions for other reasons. The use of telemetry for adequate QT interval measurement in those with baseline ECG can extend well beyond the pandemic. Therefore, telemetry as a surrogate will be helpful in preventing further exposure of healthcare workers and conserving PPE in the future.
